# Analysis on the reconstruction accuracy of the Fitch method for inferring ancestral states

**DOI:** 10.1186/1471-2105-12-18

**Published:** 2011-01-13

**Authors:** Jialiang Yang, Jun Li, Liuhuan Dong, Stefan Grünewald

**Affiliations:** 1 CAS-MPG Partner Institute for Computational Biology, Key Laboratory of Computational Biology, Shanghai Institutes for Biological Sciences, Chinese Academy of Sciences, Shanghai 200031, PR China

## Abstract

**Background:**

As one of the most widely used parsimony methods for ancestral reconstruction, the Fitch method minimizes the total number of hypothetical substitutions along all branches of a tree to explain the evolution of a character. Due to the extensive usage of this method, it has become a scientific endeavor in recent years to study the reconstruction accuracies of the Fitch method. However, most studies are restricted to 2-state evolutionary models and a study for higher-state models is needed since DNA sequences take the format of 4-state series and protein sequences even have 20 states.

**Results:**

In this paper, the ambiguous and unambiguous reconstruction accuracy of the Fitch method are studied for N-state evolutionary models. Given an arbitrary phylogenetic tree, a recurrence system is first presented to calculate iteratively the two accuracies. As complete binary tree and comb-shaped tree are the two extremal evolutionary tree topologies according to balance, we focus on the reconstruction accuracies on these two topologies and analyze their asymptotic properties. Then, 1000 Yule trees with 1024 leaves are generated and analyzed to simulate real evolutionary scenarios. It is known that more taxa not necessarily increase the reconstruction accuracies under 2-state models. The result under N-state models is also tested.

**Conclusions:**

In a large tree with many leaves, the reconstruction accuracies of using all taxa are sometimes less than those of using a leaf subset under N-state models. For complete binary trees, there always exists an equilibrium interval [*a, b*] of conservation probability, in which the limiting ambiguous reconstruction accuracy equals to the probability of randomly picking a state. The value *b *decreases with the increase of the number of states, and it seems to converge. When the conservation probability is greater than *b*, the reconstruction accuracies of the Fitch method increase rapidly. The reconstruction accuracies on 1000 simulated Yule trees also exhibit similar behaviors. For comb-shaped trees, the limiting reconstruction accuracies of using all taxa are always less than or equal to those of using the nearest root-to-leaf path when the conservation probability is not less than $\frac{1}{N}$. As a result, more taxa are suggested for ancestral reconstruction when the tree topology is balanced and the sequences are highly similar, and a few taxa close to the root are recommended otherwise.

## Background

Ancestral state reconstruction attempts to predict properties of ancestral proteins, genes and even whole genomes in a given phylogeny according to data of extant species. This approach to understanding protein functions and evolution was first proposed by Pauling and Zukerkandl in their seminal work [1]. Thereafter, with the increasing availability of biological data it has become a technique of growing importance in investigating the functions and origins of genes and proteins [2-9].

Parsimony and maximum likelihood (ML) are the two most popular criteria utilized to reconstruct ancestral states when the phylogenetic tree representing the evolutionary history of a character is known [6,10]. Parsimony methods minimize the total number of hypothetical substitutions along all branches of the evolutionary tree. The Fitch method was the first parsimony method for inferring ancestral states [11]. It is a linear time algorithm and is accurate for taxa with highly similar sequences. The method was later modified by Sankoff to account for different rates of substitutions among states [12,13]. The reader is referred to a survey book [14] for reviews of parsimony methods and their variants. In contrast to parsimony methods, ML methods choose a state to be the ancestral state such that the observed states could have been evolved from it with maximum likelihood. ML inference of ancestral sequences was pioneered by Yang, Kumar and Nei [15] and by Koshi and Goldstein [16]. Later, a widely used variant of ML method called the Bayesian approach was introduced by Huelsenbeck and his coworkers [17,18]. The reader is referred to [6] and [19] for reviews of ML methods and their variants.

Due to the extensive usage of ancestral reconstruction methods, it has become a significant scientific endeavor to study their reconstruction accuracies. These accuracies of different methods have been either estimated by statistical simulations [20,21] or calculated precisely by theoretical analyses [22-27]. For example, under a 2-state Jukes-Cantor model, several recurrence systems for calculating the reconstruction accuracies of the Fitch algorithm were presented for a given phylogenetic tree [22,24,25,27]. It was shown in these studies that the reconstruction accuracies depend largely on the topology of the phylogenetic tree. Thus, reconstruction accuracies and their asymptotic properties on the number of leaves were also analyzed for extremal trees like complete binary trees and comb-shaped trees (or rooted caterpillars) [25,27]. However, by far most theoretical analyses have been limited to 2-state models. More effort should be made to study the reconstruction accuracies under higher-state models as there are 4 states for DNA sequences and even 20 states for protein sequences.

In this paper, we study the ambiguous and unambiguous reconstruction accuracy of the Fitch algorithm for reconstructing the root state under N-state evolutionary models. We first present a general recurrence system for calculating the reconstruction accuracies on any given phylogenetic tree. We developed software that implements this system. As pointed out by Li et al. [24], more taxa are not necessarily better for the reconstruction of ancestral states. Our recurrence system and software can be used to select good subsets of taxa to reconstruct ancestral DNAs, proteins, or other characters.

After that, we restrict the analyses to 3 extremal evolutionary trees under the N-state Jukes-Cantor model, namely equal-branch complete binary tree, equal-branch comb-shaped tree and Hennigain comb-shaped tree [24,28]. It is clear that for the equal-branch trees the substitution probability along any branch is the same and is denoted by *p*, thus the conservation probability is *q*: = 1 - (*N - *1)*p*. As examples, we analyze reconstruction accuracies and their asymptotic properties on the number of leaves for *N *= 2, 4, 5, 20. We also compare the limiting ambiguous and unambiguous reconstruction accuracy of using all taxa with those of using a nearest root-to-leaf path. Finally, 1000 Yule trees with 1024 leaves are generated by the software Mesquite [29] and analyzed to simulate real phylogenetic trees.

From the studies, we observe several interesting properties for the reconstruction accuracies under *N*-state models. First, for equal-branch complete binary trees, there always exists an equilibrium interval [*a, b*] of conservation probability *q *such that, with the number of leaves tending to infinity, the ambiguous reconstruction accuracy converges to $\frac{1}{N}$, the reconstruction accuracy of randomly picking the ancestral state from *N *possible states. For example, the equilibrium interval for complete binary trees is $\left[\frac{1}{8},\frac{7}{8}\right]$ under the 2-state Jukes-Cantor model [22,25,27]. However, *a *becomes 0 when *N ≥ *3. We calculate *b *for *N *= 2, ..., 25 and find that *b *decreases slowly with the increase of *N*. The reconstruction accuracies for 1000 Yule trees exhibit similar behaviors. Second, for any equal-branch comb-shaped tree, the limiting reconstruction accuracies using the nearest root-to-leaf path are always greater than those using all taxa if the conservation probability is larger than $\frac{1}{N}$. Finally, for any Hennigian comb-shaped tree, the limiting ambiguous reconstruction accuracy is always equal to that of randomly picking a state, $\frac{1}{N}$, whereas the limiting unambiguous one is equal to $\frac{N^{N-2}}{\sum_{i=l}^{N}N^{N-i}\frac{(N-1)!}{(N-x)!}}$.

Our results suggest that more taxa should be used for reconstructing ancestral states if the tree topology is balanced and the sequences are highly similar, whereas some taxa close to the root are recommended if the tree topology is very unbalanced. In addition, under evolutionary models with molecular clock, the reconstructed state by the Fitch algorithm is as bad as a state randomly picked when the conservation probability is low or the phylogenetic tree is very unbalanced. The suggestions are also partially applicable for ML methods as ML inference of the root state is the same as that of maximum parsimony estimation under simple models such as Jukes-Cantor models when the branch lengths of the phylogenetic tree are unknown [30].

## Methods

### The Fitch Method

Let *C *be a character with state set S and *T *be a rooted phylogenetic tree in which each leaf is assigned a state in S. The Fitch method infers the states of internal nodes in a two-stage process, namely the "leaf-to-root stage" and the "root-to-leaf stage". In the first stage, it assigns a set of states *S_u _*to each node *u *of *T *as follows:

(1) If *u *is a leaf, *S_u _*contains only the state of *u*,

(2) If *u *is an internal node having children *v *and *w*,

$$S_{u}=S_{v}*S_{w}=\{\begin{array}{ll} S_{v}\cap S_{w} & \text{if}\;S_{v}\cap S_{w}\neq \emptyset ,\\ S_{v}\cup S_{w} & \text{otherwise}. \end{array}$$

The assignment starts from the leaves, and proceeds to the internal nodes until the root *r *is assigned a subset *S_r_*. In the second stage, only one state *s_u _*is chosen from the state subset *S_u _*at each node *u*. This is realized by first picking randomly a state in *S_r_*, and proceeding downwards as follows. Suppose *v *is a child of *u*, then

$$s_{v}=\{\begin{array}{ll} s_{u} & \text{if}\;s_{u}\in S_{v},\\ \text{any}\;\text{state}\;\text{in}\;S_{v} & \text{otherwise}. \end{array}$$

As a result, any state in *S_r _*is chosen as the root state with an equal probability $\frac{1}{\left|S_{r}\right|}$, where |*S_r_*| denotesthe cardinality of *S_r_*.

### Reconstruction Accuracies of the Fitch Method

Unambiguous and ambiguous reconstruction accuracy are two important criteria to evaluate the quality of a reconstruction method. Let *C *be a character with state set $S=\{s_{1},\;s_{2},\;.\;.\;.,\;s_{N}\}$. For simplicity, we order the states and restate S={1,2,⋯,N}. In order to study the reconstruction accuracies of the Fitch method on a given phylogenetic tree *T *with root *r*, a Markov model is usually assumed to represent the true biological evolutionary process of *C*, which specifies:

(1) Pr[*t_r _*= *i*], that is, the initial probability that the root state is *i *for *i *= 1, 2, ..., *N *, and

(2) Pr[*t_v _*= *j | t_u _*= *i*], that is, the transition probability that a state *i *evolves to *j *along the branch from node *u *to *v *for any *I, j *= 1, 2, ..., *N *and branch *uv*.

In particular, Pr[*t_v _*= *i | t_u _*= *i*] is called the *conservation probability *of *i *along *uv*, and Pr[*t_v _*= *j | t_u _*= *i*] with *i *≠ *j *is called the *substitution probability *from *i *to *j *along *uv*. Clearly, the probability of a state in each node is already determined by the model. For any state *i *and vertex *u *in *T*, we use Pr[*t_u _*= *i*] to denote the probability that the state of *u *is *i*. We assume throughout this paper that the evolutionary model is *symmetric *on all states, that is Pr[*t_v _*= *j | t_u _*= *i*] = Pr[*t_v _*= *i | t_u _*= *j*] and Pr[*t_v _*= *i | t_u _*= *i*] = Pr[*t_v _*= *j | t_u _*= *j*] for any two states *i *and *j*, and any branch *uv*.

After the character evolves from the root *r *under the model, the leaves will receive one of many possible distributions of the states each with some probability. The Fitch method is then applied to these distributions to infer ancestral states. Let Ψ be the set containing all the possible distributions of leaf states. For any state i∈S and any distribution D∈ Ψ, let Pr[D|i] be the probability that the leaf nodes receive the distribution D, given that the state at the root is *i*. Let B⊆S, we further let *C*(*B*, D) be the probability that the Fitch method reconstructs set *B *at root from D. Since both the evolutionary model and the Fitch method are symmetric on all states, the reconstruction accuracies are independent of the prior distribution of initial states. Thus, we choose 1 to be the root state and the *unambiguous reconstruction accuracy *(UA) is defined as

(1) $$UA=\sum_{D\in \Psi }\text{Pr}[D\;|\;1]\;C\;(\{1\},\;D),$$

the probability that it outputs the true state 1. Similarly, the *ambiguous reconstruction accuracy *(AA) is defined as

(2) $$AA=\sum_{1\in B}\frac{1}{\left|B\right|}\sum_{D\in \Psi }\text{Pr}[D|1]C(B,\;D).$$

That is, if the reconstructed set *B *contains 1, there is still a probability of $\frac{1}{\left|B\right|}$ to infer the true root state.

### Recurrence Relations to Calculate Reconstruction Accuracies

Let *Z *be an internal node with two children *X *and *Y*. Since the evolutionary model is symmetric on all *N *states, the substitution probability between any two states is the same along a given branch. We use *p_X _*and *p_Y _*to denote the substitution probabilities along branches *ZX *and *ZY*, respectively. Clearly, the corresponding conservation probabilities on any state are 1 - (*N - *1)*p_X _*and 1 - (*N - *1)*p_Y_*. In the following, we derive a recursive system involving 2*N *- 1 recursive formulas to calculate the reconstruction accuracies of a parent node from those of its two children.

Before we present the system, it is worthy of mentioning that the original dynamic programming approach in Maddison [23] can be applied to calculate both accuracies for *N*-state models. However, it involves the calculation of 2*^N ^*- 1 recursive formulas to calculate the reconstruction accuracies of a parent node from those of its two children, which is not efficient when both the evolutionary tree and the number of states *N *are large. Here, we take the advantage of the symmetries of both the Fitch method and the evolutionary models. More precisely, the reconstructed root states are categorized into 2*N *- 1 classes. Let ${ℬ}_{2i-1}=\{B\subseteq S:1\in B\text{and}\left|B\right|=i\}$ for 1 *≤ i ≤ N *and ℬ2i={B⊆S:1∈Band|B|=i} for 1 *≤ i ≤ N - *1. Then $ℬ=\{{ℬ}_{1},\ldots ,{ℬ}_{2N-1}\}$ is a partition of the set of all non-empty subsets of *S*. For any node *u*, define

$$A_{i}^{u}=\sum_{D\in \Psi }{\text{Pr}}_{u}[D|1]C_{u}({ℬ}_{i},\;D),$$

where ${\mathrm{Pr}}_{u}[D|1]$ denotes the probability that the leaf configuration under *u *is D given that the true state at *u *is 1, and $C_{u}({ℬ}_{i},D)$ denotes the probability that the reconstructed set at *u *from D is ${ℬ}_{i}$. By this definition, $UA=A_{1}^{r}$ and $AA=\sum_{k=1}^{N}\left({}_{k-1}^{N-1}\right)\frac{1}{k}A_{2k-1}^{r}$.

For any node *u*, let the reconstructed state set be *Bu*. Then for any $B\in {ℬ}_{i}$, *BZ *= *B *if and only if: (1) *B_X _*∩ *B_Y _*= *B*, or (2) *B_X _*∩ *B_Y _*= ∅ and *B_X _*∪ *B_Y _*= *B*. Thus $A_{i}^{Z}$ can be calculated from the reconstruction accuracies on node *X *and *Y *in conjunction with the substitution or conservation probabilities along the two branches *ZX *and *ZY *(see Additional File 1 for details). Recurrence formulas for 2-state models can be found in [22-27]. We present the recurrence system and initial conditions for N-state models in Additional File 1. To facilitate our study, we also implemented a computer program which takes a phylogenetic tree in Newick format and the substitution rate along branches as inputs. The phylogenetic tree can be inferred by methods like Neighbor-Joining [31] and the substitution rate can also be estimated, see for example [32]. A potential application of our algorithm and program is to select the subsets of taxa to accurately reconstruct ancestral sequences such as DNAs and proteins.

## Results and Discussion

As the reconstruction accuracies of the Fitch algorithm depend largely on the topology of phylogenetic trees, we focused our attention on two extremal tree shapes: complete binary trees which are the most balanced trees and comb-shaped trees (caterpillars), the most unbalanced trees. We are interested in trees with many taxa and in our results and figures we choose sufficiently many taxa to exhibit the asymptotic behavior. In order to simulate more realistic evolutionary scenarios, we also generated and analyzed 1000 Yule trees with 1024 leaves.

### Reconstruction Accuracies for Equal-branch Complete Binary Trees

**Figure 1:**
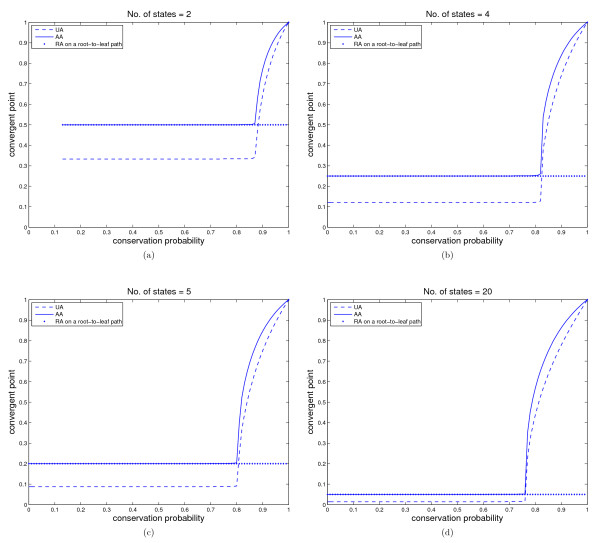
**The reconstruction accuracies from all the leaves as well as from a root-to-leaf path in an equal-branch complete binary tree ***T_n _***with ***n ***large for 2, 4, 5 and 20 states models**. UA and AA denote the unambiguous and ambiguous accuracy respectively by using all the leaves. Figure 1 (a)-(d) show the trend of reconstruction accuracies with the increase of conservation probability for the number of states 2, 4, 5 and 20, respectively. The dashed line denotes the unambiguous reconstruction accuracy by using all the leaves, the solid line denotes the ambiguous reconstruction accuracy by using all the leaves, and the diamonds the unambiguous and ambiguous accuracy by using any root-to-leaf path. Note that the unambiguous and ambiguous accuracies by any root-to-leaf path are the same.

Let *T_n _*be the equal-branch complete binary tree of 2*^n ^*leaves in which the substitution probability is *p *on each branch, and thus the conservation probability is *q *= 1 - (*N - *1)*p*. Since the subtree rooted at each child of the root *r *is the complete binary tree of 2^*n - *1 ^leaves, the recurrence system to calculate the reconstruction accuracy can be simplified (see Additional File 2). We simulated the obtained system by Matlab and studied the asymptotic properties of ambiguous and unambiguous reconstruction accuracy by using all taxa as well as by using a root-to-leaf path. The results for *N *= 2, 4, 5 and 20 are plotted in Figure 1.

Similar to the 2-state case in [24], we observed that for *N *= 4, 5 and 20: (1) UA or AA on a root-to-leaf path is always less than or equal to AA on all the terminal taxa, and (2) UA or AA on a root-to-leaf path is greater than UA on all terminal taxa when *q *is small but becomes smaller than that when q is larger than a threshold. We conjecture that the two properties hold for arbitrary number of states.

It is shown in [25,27] that for 2-state models, UA and AA diverge when $q<\frac{1}{8}$ and converge to $\frac{1}{3}$ and $\frac{1}{2}$, respectively, when $\frac{1}{8}\leq q\leq \frac{7}{8}$, as *n *tends towards infinity. Surprisingly, we observed that the divergent interval vanishes for more than two states. In addition, in an interval [0, *b*] AA converges to $\frac{1}{N}$, the probability of randomly picking a state. A possible reason is that in the 2-state scenario, the node state changes alternatingly with the increase of the level in complete binary trees. As a result, both UA and AA are alternating and thus divergent. However, their even and odd series converge to different limits (see [25,27]). For more-state models, the alternating property vanishes since there are more states to travel. If *q *is not very large, the probability for a node state to be one specific state becomes randomized as the level increases. Consequently, both UA and AA are convergent and AA converges to $\frac{1}{N}$. Clearly, *b *is a very important parameter. We listed in Table 1 the estimated values of *b *for *N *= 2, ..., 25. Table 1 shows that *b *decreases with the increase of *N *and it seems to converge to a number between 0.7 and 0.76. We offer the following explanation why more states make it easier for the Fitch algorithm to be better than random: with increasing number of states and constant conservation probability it is less likely that there are independent mutations from the root state to the same non-root state. Instead, it will happen more often that there are independent mutations from the root state to different non-root states. The latter situation makes it easier for the Fitch algorithm to reconstruct the ancestral state correctly. For example, if the reconstructed state sets at the children of an interior vertex *Z *are {1, *i*} and {1, *j*} (with *I, j *≠ 1), respectively, then the set reconstructed at *Z *is {1, *i*} if *i *= *j *and just {1} otherwise. It seems that this advantage of many states is stronger than the disadvantage of less frequent backwards mutations to the root state which are lucky for ancestral reconstruction.

**Table 1:** The estimated values of *b *for the number of states *N *= 2, ..., 25.

Estimated values of *b*								
**N**	**2**	**3**	**4**	**5**	**10**	**15**	**20**	**25**

b	0.875	0.839	0.821	0.809	0.784	0.774	0.768	0.763

Table 1 lists the estimated value of *b *by using Matlab. For convenience, we only show the values for *N *= 2, 3, 4, 5, 10, 15, 20 and 25.

In summary, when *q ≤ b *the performance of the Fitch method is as poor as randomly picking a state. Only when *q > b*, the Fitch method could be used to reconstruct ancestral states and the performance improves quickly with the increase of *q*. As a conclusion, conservation probability is the most important factor to determine the performance of the Fitch method. The method is reliable only when *q *is large, which indicates that the taxa are highly similar. However, as we know, when taxa are not similar, no reconstruction method performs good, so more effort should be made in developing a reliable method in this scenario. A suggestion for ancestral reconstruction is that, instead of treating all taxa as a whole, one first reconstructs subset of very similar taxa and make use of the reconstructed ancestral sequences to infer the ancestor of the whole taxa set.

### Reconstruction Accuracies on Comb-shaped Trees

**Figure 2:**
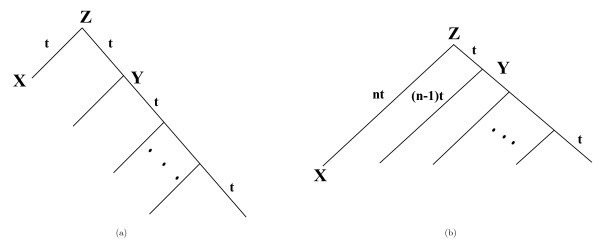
**Two kinds of comb-shaped trees**. Figure 2 shows two kinds of comb-shaped trees: (a) an equal-branch comb-shaped tree, (b) a Hennigian comb-shaped tree with *n *leaves.

Due to their extreme unbalanceness, comb-shaped trees (or rooted caterpillars) are widely studied. A comb-shaped tree is a rooted binary tree where every internal node is adjacent to at least one leaf node. We studied two comb-shaped trees as shown in Figure 2. The tree in Figure 2 is called *equal-branch comb-shaped tree*, which assumes that substitution happens mostly at speciation events and thus each branch length can be considered as equal. The other tree is called *Hennigian comb-shaped tree*, which assumes that substitution occurs continuously during the course of evolution. More precisely, we assume that all interior edges have the same length and all leaves are equally far away from the root.

#### Equal-branch Comb-shaped Trees

**Figure 3:**
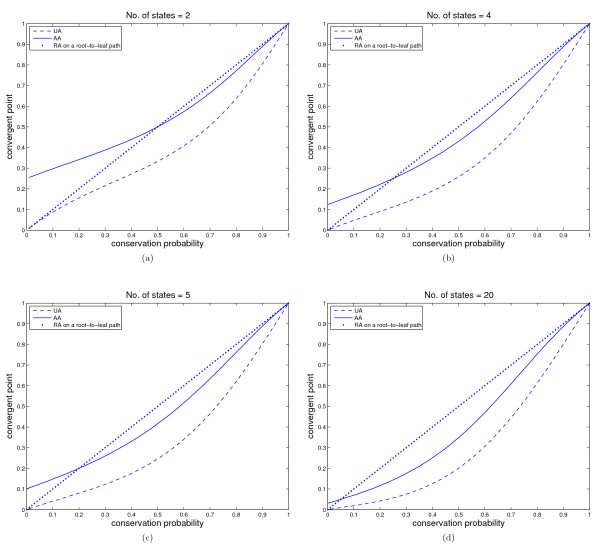
**The reconstruction accuracies from all the leaves as well as from a root-to-leaf path in an equal-branch comb-shaped tree ***T_n _***with ***n ***large for 2, 4, 5 and 20 states models**. Figure 3 (a)-(d) show the trend of reconstruction accuracies with the increase of conservation probability for the number of states 2, 4, 5 and 20 respectively. The dashed line denotes the unambiguous reconstruction accuracy by using all the leaves, the solid line denotes the ambiguous reconstruction accuracy by using all the leaves, and the diamonds the unambiguous and ambiguous accuracy by using the nearest root-to-leaf path. Note that the unambiguous and ambiguous accuracies by the nearest root-to-leaf path are the same.

In this tree, a descendant leaf *X *of the root is closer in evolutionary distance than other leaves. Let the substitution probability of any kind along any branch be *p*. Since the left subtree of the root is a branch and the right subtree is a repeat of the tree with one leaf less, the recurrence formula can be obtained by substituting $A_{i}^{Z}=A_{i}^{n},A_{i}^{Y}=A_{i}^{n-1}$ for $i=1,\cdots ,2N-1,A_{1}^{X}=1$ and $A_{j}^{X}=0$ for *j *≠ 1. We simulated the obtained system by Matlab and studied the asymptotic properties. The results for *N *= 2, 4, 5 and 20 are plotted in Figure 3.

An interesting observation is that, in contrast to complete binary trees, there is no interval of conservation probability such that AA converges to $\frac{1}{N}$ for any equal-branch comb-shaped tree. A possible reason is that the leaves that are close to the root dominate the reconstruction accuracy and their distances to the root do not increase with time. In addition, the limiting AA using the nearest root-to-leaf path is always greater than that using all taxa if conservation probability is larger than $\frac{1}{N}$.

#### Hennigian Comb-shaped Trees

Clearly, complete binary tree and Hennigian comb-shaped tree are the two kinds of extremal ultrametric trees. By comparing the reconstruction accuracies on both trees with those on real evolutionary trees, one can examine which extremal trees are more realistic. Under the Jukes-Cantor model, for a Hennigian comb tree in which each branch has its own length *l*, the substitution probability is $p=\frac{1}{N}-\frac{1}{N}e^{-N\lambda l}$, and the conservation probability is $q=1-(N-1)p=\frac{1}{N}+\frac{N-1}{N}e^{-N\lambda l}$, where *λ *is the substitution rate. Similarly, the recurrence system to calculate UA of the Fitch algorithm along the Hennigian tree can be derived from the general recurrence relations.

An observation from the recurrence system is that AA is always convergent to $\frac{1}{N}$. As an indication, the Fitch algorithm is not suitable for ancestral reconstruction on evolutionary trees with shape similar to Hennigian comb-shaped trees with many taxa. In addition, UA is convergent to $\frac{N^{N-2}}{\sum_{i=l}^{N}N^{N-i}\frac{(N-1)!}{(N-x)!}}$ (See Additional File 3 for details). To illustrate the result, we also listed in Table 2 the simulated convergent values of UA from the recurrence system for some numbers *N *between 2 and 50.

**Table 2:** The estimated values for UA of Hennigian comb-shaped trees when *n *is large for the number of states *N *= 2, ..., 50.

Estimated values of the limiting UA for Hennigian trees										
**N**	**2**	**3**	**4**	**5**	**10**	**15**	**20**	**30**	**40**	**50**

UA	0.3333	0.1765	0.1126	0.0797	0.0274	0.0145	0.0098	0.0053	0.0034	0.0022

Table 2 lists the estimated value of UA of Hennigian trees when *n *is large by using Matlab. For convenience, we only show the values for *N *= 2, 3, 4, 5, 10, 15, 20, 30, 40 and 50.

### Reconstruction Accuracies for Yule Trees with 1024 Leaves

**Figure 4:**
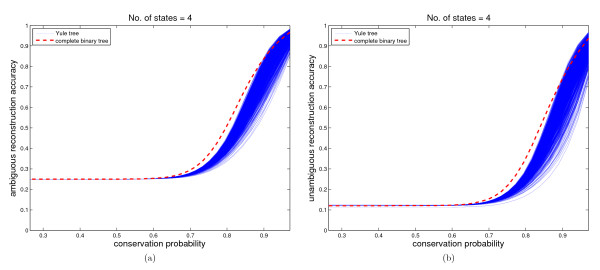
**The comparison of unambiguous and ambiguous reconstruction accuracy between 1000 randomly generated Yule trees and the equal-branch complete binary tree with 1024 leaves**. Figure 4 (a)-(b) show the trend of ambiguous and unambiguous reconstruction accuracies with the increase of conservation probability for 1000 randomly generated Yule trees and the equal-branch complete binary tree with 1024 leaves. The dashed line denotes the trend of reconstruction accuracy on the equal-branch complete binary tree, the solid lines denote the trend of reconstruction accuracy on the randomly generated Yule trees.

As can be seen from Figure 4, the 1000 randomly generated Yule trees show the same qualitative behavior as the equal-branch complete binary tree with the same number of leaves. That is, there is an interval [0, *b*] of conservation probability *q *in which AA is almost equal to the probability of randomly picking a state, when *q > b*, the reconstruction accuracies are improved quickly with the increase of *q*. A possible explanation is that the number of taxa increases exponentially with the time, and correspondingly, very long edges are rare. Another interesting observation is that sometimes the reconstruction accuracies on the Yule trees are even higher than those of the complete binary tree. This contradicts the intuition that equal-branch complete binary trees always maximize the reconstruction accuracies and leaves an open problem which trees do so.

## Conclusions

In this paper, we study the unambiguous and ambiguous reconstruction accuracy of the Fitch method. We first present a general recurrence system as well as a program for calculating reconstruction accuracies on arbitrary trees. Based on the system and program, we analyze 3 special trees under the Jukes-Cantor evolutionary model, namely equal-branch complete binary trees, equal-branch comb-shaped trees, and Hennigian comb-shaped trees, as well as 1000 randomly generated Yule trees to simulate real evolutionary scenarios. From the analyses, we conclude that (1) for equal-branch complete binary trees, there always exists an interval [0, *b*] of conservation probability, in which the ambiguous reconstruction probability converges to $\frac{1}{N}$, the probability of randomly picking a state, when the conservation probability is greater than *b*, both reconstruction accuracies increase rapidly, The randomly generated Yule trees also exhibit the same behavior, (2) For unbalanced trees like comb-shaped trees, the reconstruction accuracies using the nearest root-to-leaf path are always greater than or equal to those using all taxa. As a conclusion, more taxa are suggested for ancestral reconstruction when the tree topology is balanced and the sequences of taxa are highly similar, and a few taxa close to the root are recommended otherwise.

## Availability and Requirements

The software as well as the source code in C_++ _to calculate the reconstruction accuracy of the Fitch method on any tree with arbitrary states under the one parameter Jukes-Cantor model can be found in Additional File 4. The reader is referred to the "install.txt" and "help.txt" file for the installation and usage of the program, or alternatively run the bash file "accuracy.out" in a Unix/Linux system. The programs to draw the figures and tables are written in Matlab, which can also be found in Additional File 4. So Matlab should be installed to run these codes.

## Authors' contributions

SG and JY conceived the methods. JL and JY performed the experiments. JY prepared the manuscript. All authors contributed to the discussion and have approved the final manuscript.

## Supplementary Material

Additional file 1 **The recurrence system for the reconstruction accuracy of the Fitch method on ***N***-state models**. In this file, we provide the general recurrence system and initial conditions for calculating the reconstruction accuracy of the Fitch method on *N*-state models. Click here for file

Additional file 2 **The recurrence system of calculating reconstruction accuracies for the complete binary tree ***T_n_*. In this file, we provide the recurrence system and initial conditions for calculating the reconstruction accuracy of the Fitch method on the complete binary tree *T_n _*with 2*^n ^*leaves. Click here for file

Additional file 3 **A sketch of the proof for the formula of limiting UA on Hennigian comb-shaped trees**. In this file, we provide a sketch of the proof that the limiting UA on Hennigian comb-shaped trees with *N *leaves is $\frac{N^{N}}{\sum_{i=l}^{N}N^{N-i}\frac{N!}{(N-x)!}}$. Click here for file

Additional file 4 **Programs and source codes to calculate the reconstruction accuracy and draw figures and tables**. In this file, we present the software as well as its source code in C_++ _to calculate the reconstruction accuracy of the Fitch method on any tree with arbitrary states under the one parameter Jukes-Cantor model. The programs in Matlab to draw the figures and tables for extremal trees are also provided. Click here for file
