# Supplementary material for: Analysis on the reconstruction accuracy of the Fitch method for inferring ancestral states
Source: BMC Bioinformatics. 2011 Jan 13;12:18. doi: 10.1186/1471-2105-12-18 (PMC3030536; doi:10.1186/1471-2105-12-18)
Supplement: Additional file 1 — The recurrence system for the reconstruction accuracy of the Fitch method on N-state models. In this file, we provide the general recurrence system and initial conditions for calculating the reconstruction accuracy of the Fitch method on N-state models. [file 1471-2105-12-18-S1.PDF]

Additional File 1 — The recurrence system for the reconstruction accuracy of the Fitch method on N-state models

Let $\mathcal{S} = \{1, 2, \cdots, N\}$ be the set of character states. For any node u, let $s \in \mathcal{S}$ be the true state at u, $\mathcal{D}'$ be any state configuration of leaves below u evolving from s, and Ψ' be the set of all such configurations. We further use $\Pr_u[\mathcal{D}' \mid s]$ to denote the probability that the leaf configuration under u is $\mathcal{D}'$ given that the true state at u is s, and $C_u(B, \mathcal{D}')$ to denote the probability that the reconstructed set at u from $\mathcal{D}'$ is B for any $B \subseteq \mathcal{S}$. Then the probability that the reconstructed state set at u is B, given that the true state is s, can be defined as

$$\Pr_u[B|s] = \sum_{\mathcal{D} \in \Psi'} \Pr_u[\mathcal{D} \mid s] C_u(B, \mathcal{D}).$$

Since both the evolutionary model and the Fitch method are symmetric on all states, we have the following properties:

1. For any $B_s \subseteq S$ and $s \neq 1 \in S$, there exists $1 \in B_1 \subseteq S$ with $|B_s| = |B_1|$, such that

   $$\Pr_u[B_s|s] = \Pr_u[B_1|1].$$

   For example, $\Pr_u[\{2\}|2] = \Pr_u[\{1\}|1]$, $\Pr_u[\{2,3\}|2] = \Pr_u[\{1,2\}|1]$ and $\Pr_u[\{1,3\}|2] = \Pr_u[\{2,3\}|1]$. Thus, we can always assume 1 to be the true state at u.

2. There are only two different probabilities for each reconstructed sets with the same cardinality, that is, the true state is in the set or not. For example, $\Pr_u[\{1,2,3\}|1] = \Pr_u[\{1,4,20\}|1]$, but might not equal to $\Pr_u[\{2,3,4\}|1]$. Thus, we categorize all state sets into $2N-1$ classes. Specifically, we define $\mathcal{B}_{2i-1} = \{B \subseteq \mathcal{S} : 1 \in B \text{ and } |B| = i\}$ for $1 \le i \le N$ and $\mathcal{B}_{2i} = \{B \subseteq \mathcal{S} : 1 \notin B \text{ and } |B| = i\}$ for $1 \le i \le N-1$. Then $\mathcal{B} = \{\mathcal{B}_1, \cdots, \mathcal{B}_{2N-1}\}$ is a partition of the set of all non-empty subsets of $\mathcal{S}$.

For $1 \le i \le 2N-1$ and any $B \in \mathcal{B}_i$, define

$$A_i^u = \Pr_u[B|1].$$

By this definition, $UA = A_1^r$ and $AA = \sum_{k=1}^{N} \binom{N-1}{k-1} \frac{1}{k} A_{2k-1}^r$, and for any leaf l, $A_1^l = 1$ and $A_i^l = 0$ for $2 \le i \le 2N-1$.

We next derive a recurrence system to calculate A_i^r for $1 \le i \le 2N-1$ from leaves. Let Z be an internal node with two children X and Y. We calculate A_i^Z from X and Y in a similar fashion to Maddison [1].

For any node u, let the reconstructed state set be R_u and the true state be t_u. By definition $A_i^Z = \Pr_Z[B|1]$ with $B \in \mathcal{B}_i$. However, $R_Z = B$ if and only if: (1) $R_X \cap R_Y = B$; or (2) $R_X \cap R_Y = \emptyset$ and $R_X \cup R_Y = B$. Thus

$$\begin{aligned} A_i^Z &= \Pr_Z[B|1] = \Pr_Z[\{\bigcup_{R_X \cap R_Y = B} R_X \cap R_Y\}|1] + \Pr_Z[\{\bigcup_{R_X \cap R_Y = \emptyset,\ R_X \cup R_Y = B} R_X \cap R_Y\}|1] \\ &= \sum_{R_X \cap R_Y = B} \Pr_Z[R_X|1]\Pr_Z[R_Y|1] + \sum_{R_X \cap R_Y = \emptyset,\ R_X \cup R_Y = B} \Pr_Z[R_X|1]\Pr_Z[R_Y|1], \end{aligned}$$

since given the true state at Z is 1, R_X and R_Y are independent. Thus, we only need to calculate $\Pr_Z[R_X|1]$. Noticing that,

$$\begin{aligned} \Pr_Z[R_X|1] &= \Pr_Z[R_X \cap \{\bigcup_{i=1}^{N} t_X = i\} \mid t_Z = 1] \\ &= \sum_{i=1}^{N} \Pr_X[R_X|t_X = i] \Pr[t_X = i \mid t_Z = 1], \end{aligned}$$

where $\Pr[t_X = i \mid t_Z = 1]$ equals to p_X if $i \neq 1$, and $1-(N-1)p_X$ otherwise; and $\Pr_X[R_X|t_X = i]$ equals to $A^X_{2|R_X|-1}$ if $i \in R_X$, and $A^X_{2|R_X|}$ otherwise.

Using the above technique, we arrive at the following recurrence system. For $1 \leq k \leq N$,

$$\begin{aligned} A_{2k-1}^Z = & \sum_{i=0}^{N-k} \binom{N-k}{i} \Big\{[1-(N-i-k)p_X]A^X_{2(i+k)-1} + (N-i-k)p_X A^X_{2(i+k)}\Big\} \\ & \left\{ \sum_{j=0}^{N-i-k} \binom{N-i-k}{j} \Big\{[1-(N-j-k)p_Y]A^Y_{2(j+k)-1} + (N-j-k)p_Y A^Y_{2(j+k)}\Big\} \right\} \\ & + \sum_{i=0}^{k-2} \binom{k-1}{i} \Big\{[1-(N-i-1)p_X]A^X_{2i+1} + (N-i-1)p_X A^X_{2i+2}]\Big\} \\ & \Big\{(k-i-1)p_Y A^Y_{2(k-i-1)-1} + [1-(k-i-1)p_Y]A^Y_{2(k-i-1)}\Big\} \\ & + \sum_{i=0}^{k-2} \binom{k-1}{i} \Big\{(k-i-1)p_X A^X_{2(k-i-1)-1} + [1-(k-i-1)p_X]A^X_{2(k-i-1)}\Big\} \\ & \Big\{[1-(N-i-1)p_Y]A^Y_{2i+1} + (N-i-1)p_Y A^Y_{2i+2}]\Big\}; \end{aligned}$$

for $1 \le k \le N-1$,

$$A^Z_{2k} = \sum_{i=0}^{N-k-1} \binom{N-k-1}{i} \left\{ (i+k)p_X A^X_{2(i+k)-1} + [1-(i+k)p_X] A^X_{2(i+k)} \right\}$$
$$\left\{ \sum_{j=0}^{N-i-k-1} \binom{N-i-k-1}{j} \left\{ (j+k)p_Y A^Y_{2(j+k)-1} + [1-(j+k)p_Y] A^Y_{2(j+k)} \right\} \right.$$
$$\left. + \sum_{j=0}^{N-i-k-1} \binom{N-i-k-1}{j} \left\{ [1-(N-j-k-1)p_Y] A^Y_{2(j+k)+1} + (N-j-k-1)p_Y A^Y_{2(j+k)+2} \right\} \right\}$$
$$+ \sum_{i=0}^{N-k-1} \binom{N-k-1}{i} \left\{ [1-(N-i-k-1)p_X] A^X_{2(i+k)+1} + (N-i-k-1)p_X A^X_{2(i+k)+2} \right\}$$
$$\left\{ \sum_{j=0}^{N-i-k-1} \binom{N-i-k-1}{j} \left\{ (j+k)p_Y A^Y_{2(j+k)-1} + [1-(j+k)p_Y] A^Y_{2(j+k)} \right\} \right\}$$
$$+ \sum_{i=1}^{k-1} \binom{k}{i} \left\{ ip_X A^X_{2i-1} + (1-ip_X) A^X_{2i}] \right\} \times \left\{ (k-i)p_Y A^Y_{2(k-i)-1} + [1-(k-i)p_Y] A^Y_{2(k-i)} \right\},$$

and

$$\sum_{k=1}^{N} \binom{N-1}{k-1} A^Z_{2k-1} + \sum_{k=1}^{N-1} \binom{N-1}{k} A^Z_{2k} = 1,$$

with initial conditions $A^l_1 = 1$ and $A^l_i = 0$ for $i = 2, \cdots, 2N-1$ and any leaf node l. In the above system, we let $A^Z_{2N} = A^Z_{2N-1}$ for convenience.

References

[1] Maddison W: **Calculating the probability distributions of ancestral states reconstructed by parsimony on phylogenetic trees.** *Syst. Biol.* 1995, **44:** 474-481.
