# Supplementary material for: Analysis on the reconstruction accuracy of the Fitch method for inferring ancestral states
Source: BMC Bioinformatics. 2011 Jan 13;12:18. doi: 10.1186/1471-2105-12-18 (PMC3030536; doi:10.1186/1471-2105-12-18)
Supplement: Additional file 2 — The recurrence system of calculating reconstruction accuracies for the complete binary tree Tn. In this file, we provide the recurrence system and initial conditions for calculating the reconstruction accuracy of the Fitch method on the complete binary tree Tn with 2n leaves. [file 1471-2105-12-18-S2.PDF]

Additional File 2 — The recurrence system of calculating reconstruction accuracies for the complete binary tree T_n

We use A_k^n to denote A_k^Z, where there are 2^n leaves below Z, the root of T_n. Then $A_k^X = A_k^Y = A_k^{n-1}$. Thus, from the general recurrence system, we have for $1 \le k \le N$

$$
\begin{aligned}
A_{2k-1}^n \quad = \quad & \sum_{i=0}^{N-k} \binom{N-k}{i} \left\{ [1-(N-i-k)p] A_{2(i+k)-1}^{n-1} + (N-i-k)p A_{2(i+k)}^{n-1} \right\} \\
& \left\{ \sum_{j=0}^{N-i-k} \binom{N-i-k}{j} \left\{ [1-(N-j-k)p] A_{2(j+k)-1}^{n-1} + (N-j-k)p A_{2(j+k)}^{n-1} \right\} \right\} \\
& + \sum_{i=0}^{k-2} \binom{k-1}{i} \left\{ [1-(N-i-1)p] A_{2i+1}^{n-1} + (N-i-1)p A_{2i+2}^{n-1}] \right\} \\
& \left\{ (k-i-1)p A_{2(k-i-1)-1}^{n-1} + [1-(k-i-1)p] A_{2(k-i-1)}^{n-1} \right\} \\
& + \sum_{i=0}^{k-2} \binom{k-1}{i} \left\{ (k-i-1)p A_{2(k-i-1)-1}^{n-1} + [1-(k-i-1)p] A_{2(k-i-1)}^{n-1} \right\} \\
& \left\{ [1-(N-i-1)p] A_{2i+1}^{n-1} + (N-i-1)p A_{2i+2}^{n-1}] \right\};
\end{aligned}
$$

for $1 \le k \le N-1$

$$
\begin{aligned}
A_{2k}^n \quad = \quad & \sum_{i=0}^{N-k-1} \binom{N-k-1}{i} \left\{ (i+k)p A_{2(i+k)-1}^{n-1} + [1-(i+k)p] A_{2(i+k)}^{n-1} \right\} \\
& \left\{ \sum_{j=0}^{N-i-k-1} \binom{N-i-k-1}{j} \left\{ (j+k)p A_{2(j+k)-1}^{n-1} + [1-(j+k)p] A_{2(j+k)}^{n-1} \right\} \right. \\
& \left. + \sum_{j=0}^{N-i-k-1} \binom{N-i-k-1}{j} \left\{ [1-(N-j-k-1)p] A_{2(j+k)+1}^{n-1} + (N-j-k-1)p A_{2(j+k)+2}^{n-1} \right\} \right\} \\
& + \sum_{i=0}^{N-k-1} \binom{N-k-1}{i} \left\{ [1-(N-i-k-1)p] A_{2(i+k)+1}^{n-1} + (N-i-k-1)p A_{2(i+k)+2}^{n-1} \right\} \\
& \left\{ \sum_{j=0}^{N-i-k-1} \binom{N-i-k-1}{j} \left\{ (j+k)p_{n-1} A_{2(j+k)-1}^{n-1} + [1-(j+k)p] A_{2(j+k)}^{n-1} \right\} \right\} \\
& + \sum_{i=1}^{k-1} \binom{k}{i} \left\{ ip A_{2i-1}^{n-1} + (1-ip_X) A_{2i}^{n-1}] \right\} \times \left\{ (k-i)p A_{2(k-i)-1}^{n-1} + [1-(k-i)p] A_{2(k-i)}^{n-1} \right\},
\end{aligned}
$$

where we let $A_{2N} = A_{2N-1}$; and further

$$
\sum_{k=1}^{N} \binom{N-1}{k-1} A_{2k-1}^n + \sum_{k=1}^{N-1} \binom{N-1}{k} A_{2k}^n = 1,
$$

with initial conditions $A_1^0 = 1$ and $A_i^0 = 0$ for $i = 2, \cdots, 2N-1$.
