# Supplementary material for: Analysis on the reconstruction accuracy of the Fitch method for inferring ancestral states
Source: BMC Bioinformatics. 2011 Jan 13;12:18. doi: 10.1186/1471-2105-12-18 (PMC3030536; doi:10.1186/1471-2105-12-18)
Supplement: Additional file 3 — A sketch of the proof for the formula of limiting UA on Hennigian comb-shaped trees. In this file, we provide a sketch of the proof that the limiting UA on Hennigian comb-shaped trees with N leaves is NN∑i=lNNN−iN!(N−x)!. [file 1471-2105-12-18-S3.PDF]

Additional File 3 — A sketch of a proof for the formula of limiting UA on Hennigian comb-shaped trees

For a Hennigan comb shaped tree, let Z be an internal node, under which the number of leaves is large. Let X and Y be the left and right children of Z, thus X is a leaf and Y is a root of a smaller comb-shaped tree. Suppose that the branch length of ZX tends towards infinity, then by the Jukes-Cantor model the true state at X is randomized, that is, $\Pr_X[t_X = i] = \frac{1}{N}$ for $i \in \mathcal{S}$, where t_X denotes the true state at X.

Let i be the cardinality of the set of states that the Fitch algorithm reconstructs at Y. Then the reconstructed set at Z contains either $i+1$ or 1 state(s), with probability $\frac{N-i}{N}$ and $\frac{i}{N}$, respectively. As a result, the cardinalities of the reconstructed state set by the Fitch algorithm from leaves to the root can be formulated by a Markov process, in which the state set is $\{1, 2, \cdots, N\}$ and the transition matrix is

$$T = \begin{bmatrix} \frac{1}{N} & \frac{N-1}{N} & 0 & \cdots & 0 \\ \frac{2}{N} & 0 & \frac{N-2}{N} & \cdots & 0 \\ \vdots & \vdots & \vdots & \vdots & \vdots \\ \frac{N-1}{N} & 0 & 0 & \cdots & \frac{1}{N} \\ 1 & 0 & 0 & 0 & 0 \end{bmatrix} \tag{1}$$

For every pair of states i and j, there is a walk $i, \overbrace{1, 1, \cdots, 1}^{N-j+1}, 2, 3, \cdots, j-1, j$ from i to j of length N with non-zero probability. Thus, the transition matrix T is primitive. By the Perron-Frobenius theorem [1], there exists an equilibrium state vector $\vec{v} = (v_1, v_2, \cdots, v_N)$, such that $\vec{v} = \vec{v}T$. Since the state at X is randomized, the probability that the reconstructed cardinality 1 set contains the true state is $\frac{1}{N}$. Thus, the limiting UA is $\frac{1}{N}v_1$, which is $\frac{N^{N-2}}{\sum_{i=1}^{N} N^{N-i}\frac{(N-1)!}{(N-i)!}}$.

References

[1] Perron, O: **Zur Theorie der Matrices**. *Mathematische Annalen* 1907, **64(2):** 248-263, doi:10.1007/BF01449896.
